# *Staphylococcus aureus* with inducible clindamycin resistance and methicillin resistance in a tertiary hospital in Nepal

**DOI:** 10.1186/s41182-021-00392-2

**Published:** 2021-12-27

**Authors:** Devi Thapa, Susil Pyakurel, Sabita Thapa, Suresh Lamsal, Mahesh Chaudhari, Nabaraj Adhikari, Dhiraj Shrestha

**Affiliations:** 1 Department of Microbiology, Kantipur College of Medical Science, Kathmandu, Nepal; 2 Department of Microbiology, Shi-Gan International College of Science and Technology, Kathmandu, Nepal; 3 Department of Microbiology, KIST Medical College, Kathmandu, Nepal; 4 Central Department of Microbiology, Tribhuvan University, Kirtipur, Nepal

**Keywords:** Inducible clindamycin resistance, MRSA, Nepal, *S. aureus*

## Abstract

**Background:**

*Staphylococcus aureus* is a global public health issue in both community and hospital settings. Management of methicillin-resistant *S. aureus* (MRSA) infections are tough owing to its resistance to many antibiotics. Macrolide-lincosamide-streptogramin B (MLSB) antibiotics are commonly used for the management of MRSA. This study was aimed to determine the occurrence of inducible clindamycin- and methicillin-resistant *S. aureus* at a tertiary care hospital in Kathmandu, Nepal.

**Methods:**

A total of 1027 clinical samples were processed following standard laboratory procedures and antibiotic susceptibility testing of *S. aureus* was performed by disc diffusion method. MRSA isolates were detected phenotypically using cefoxitin disc, and inducible clindamycin resistance was detected phenotypically using the D-zone test.

**Results:**

Of 1027 samples, 321 (31.2%) were culture positive, of which 38 (11.8%) were *S. aureus*. All *S. aureus* isolates were susceptible to vancomycin, and 25 (67%) of *S. aureus* isolates were multidrug-resistant. Similarly, 15 (39.5%) of *S. aureus* were MRSA and 14 (36.5%) were inducible clindamycin-resistant phenotypes.

**Conclusion:**

Inducible clindamycin and methicillin resistance were common in *S. aureus*. This emphasizes that the methicillin resistance test and the D-zone test should be incorporated into the routine antibiotic susceptibility testing in hospital settings.

## Introduction

Globally, *Staphylococcus aureus* is a leading cause of nosocomial and community-acquired infections [1]. The rampant use of antibiotics has increased the selective pressure on bacteria, resulting in the emergence of drug resistance. These resistant bacteria pose a global public health threat [2]. The dissemination and spread of resistant isolates reduce the efficacy of antimicrobial agents which in turn prolongs hospital stays, increases treatment costs, and increases fatalities [3]. One of the common resistance mechanisms in *S. aureus* is methicillin resistance. The methicillin resistance was first reported in 1961, just 2 years after the first clinical use of methicillin [4]. Since then, the rapid rise of methicillin-resistant *S. aureus* (MRSA) has limited the therapeutic choices for the management of MRSA infections [5].

Antibiotics like vancomycin, linezolid, quinupristin, and dalfopristin have long been the preferred choice for the management of such MRSA isolates. However, the increasing reports of resistance against these antibiotics have only increased the skepticism on their efficacy [6, 7]. This suspicion has led clinicians to choose the macrolide lincosamide-streptogramin B (MLSB) family of antibiotics, a reserved alternative, for the management of MRSA isolates. In the MLSB family, a commonly used clindamycin is an ideal antibiotic owing to its excellent pharmacokinetics [8]. With time and overuse, *S. aureus* is acquiring resistance against MLSB too. Resistance against MSLB antibiotics can be either constitutive or inducible. The constitutive resistance mechanism is mediated through msrA genes, in which *S*. aureus strains are resistant to erythromycin and sensitive to clindamycin, in both in vivo and in vitro. The constitutively resistant strains do not develop clindamycin resistance during therapy [9, 10]. The inducible MLSB (iMLSB) resistant isolates show resistance against erythromycin but are susceptible to clindamycin. In the presence of a powerful methylase enzyme inducer like erythromycin, iMLSB resistance develops. Unlike constitutive MLSB (cMLSB) resistance, iMLSB resistance cannot be detected by standard susceptibility testing. The inducible clindamycin resistance can be detected by the D-zone test, i.e. D-shaped distorted inhibition zone around clindamycin under the in-vitro effect of erythromycin [11]. It is critical to identify the iMLSB resistance for the proper management of *S. aureus* [12]. Otherwise, clindamycin administration can lead to treatment failure by the development of constitutive resistance [13].

The prevalence of methicillin and inducible clindamycin resistance among *S. aureus* varies considerably as per settings and regions. Various studies have reported the prevalence of MRSA in the world ranging from 20 to 58% [9, 11, 12, 14–19]. Few studies have reported MRSA in Nepal ranging from 25 to 64% [20–25]. Similarly, various studies had reported prevalence of inducible clindamycin resistance among *S. aureus* in the world ranging from 7 to 34% [1, 9–12, 14–19]. Few studies have reported inducible clindamycin resistance among *S. aureus* in Nepal ranging from 11 to 40% [20–26]. However, it is largely under-reported as there are limited studies on the detection of methicillin and inducible clindamycin resistance in *S. aureus*. Ironically, the detection of methicillin and inducible clindamycin resistance are still not a part of routine susceptibility investigations of *S. aureus* in hospital settings. The local resistance data is crucial for optimizing antibiotics usage, guiding empirical treatment, and managing infection effectively. The study aimed to explore the burden of methicillin and inducible clindamycin resistance among *S. aureus* in a tertiary care hospital in Nepal. Since antimicrobial susceptibility among pathogens is dynamics of time and space, findings from such surveillance study will assist clinicians of the region in the jurisdiction of appropriate antibiotics and improve clinical management of infections.

## Methods

### Study design, study area, and sample population

This cross-sectional study was carried out from August 2018 to March 2019 at Kantipur Hospital in Kathmandu, Nepal. The hospital is a 100-bed referral hospital located at the Eastern gate of the capital city, Kathmandu. The hospital serves patients of the Kathmandu valley and other nearby areas. Also, the hospital serves patients referred to from other hospitals outside the Kathmandu valley, especially those from Eastern region of the country. Kathmandu has a total area of 50.67 sq. km and has an average elevation of 1400 m above sea level. The population density is approximately 4416 per sq. km. The sample population included patients of all age groups and genders visiting the hospital during the study period with symptoms of suspected infections. Both inpatients and outpatients were included in the study. The demographic information of the patients was retrieved from the medical records of the hospital. The samples from the patients were collected following the physician’s clinical diagnosis. A total of 1027 clinical samples were included in the study. The samples included urine (*n* = 859), pus (*n* = 52), blood (*n* = 50), sputum (*n* = 41), and body fluids (*n* = 25; CSF, synovial fluid, pleural fluid, throat swabs, vaginal swabs). Repeated samples and samples showing the possible signs of contaminations were excluded.

### Sample processing and identification of *S. aureus*

All the samples were cultured in a routine culture media following the standard microbiological protocols. In brief, the samples were first streaked on blood agar and mannitol salt agar. Then, the mannitol salt agar plates were incubated aerobically at 37 °C for 24 h, while blood agar plates were incubated in a candle jar at 37 °C for 24 h. Plates yielding cream to golden yellow colonies with or without hemolysis on blood agar, and yellow colonies on mannitol salt agar were subcultured in nutrient agar [27, 28]. *S. aureus* isolates were identified based on growth in culture, Gram’s staining reactions (Gram-positive cocci in a cluster), and various biochemical properties (catalase-positive, coagulase-positive, DNase-positive, nitrate reduction-positive, and glucose OF-fermentative) [28].

### Antibiotic susceptibility testing

All identified isolates of *S. aureus* were tested for susceptibility against the commercially available antibiotic discs by modified Kirby-Bauer disc diffusion method on Mueller–Hinton agar (MHA) (HiMedia Pvt. Ltd., India), as described in CLSI M100-S28 [29]. In brief, 0.5 McFarland bacterial suspensions were prepared from a single isolated colony on the nutrient broth. Lawn cultures of this bacterial suspension were performed over the MHA plate. Subsequently, antibiotic discs were placed on the agar surface using sterile forceps. Finally, the plates were incubated aerobically at 37 °C for 24 h [29]. The antibiotic discs tested were amikacin (30 μg), ampicillin (10 μg), cefoxitin (30 μg), ceftriaxone (30 μg), ciprofloxacin (5 μg), clindamycin (2 μg), co-trimoxazole (sulpha/trimethoprim) (23.75/ 1.25 μg), erythromycin (15 μg), gentamicin (10 μg), norfloxacin (10 μg), oxacillin (1 μg), penicillin-G (10 μg), tetracycline (30 μg), vancomycin (Etest for MIC) (HiMedia Pvt. Ltd., India). The zone diameters were interpreted as per CLSI M100-S28 [29]. The CLSI recommends interpreting antibiotic susceptibility results as resistant (R), or intermediate (I), or susceptible (S), but we dichotomously categorized them as susceptible (S) or resistant (R). The isolates showing intermediate susceptibility were also considered resistant. Quality control was maintained using *S. aureus* ATCC 25923. The tested antibiotics were categorized into nine classes viz., aminoglycoside, cephems, fluoroquinolone, folate pathway inhibitor, glycopeptide, lincosamide, macrolide, penicillin, and tetracycline. *S. aureus* resistant to three or more different classes of antibiotics were considered multidrug-resistant (MDR) [30].

### Phenotypic detection of MRSA

The MRSA isolates were confirmed phenotypically using cefoxitin disc (30 μg) (HiMedia Pvt. Ltd., India). As recommended by CLSI M100-S28, cefoxitin disc was used as a surrogate for detection of *mec*A-mediated oxacillin resistance, i.e. MRSA, by disc diffusion method. The plates were incubated aerobically at 33 to 35 °C for 18 h *S. aureus* yielding zone diameter of 21 mm or less with cefoxitin disc was phenotypically confirmed as MRSA, as per CLSI M100-S28 [29]. *S. aureus* ATCC 25923 was used as quality control.

### Phenotypic detection of inducible clindamycin resistance (iMLSB phenotypes)

**Fig. 1:**
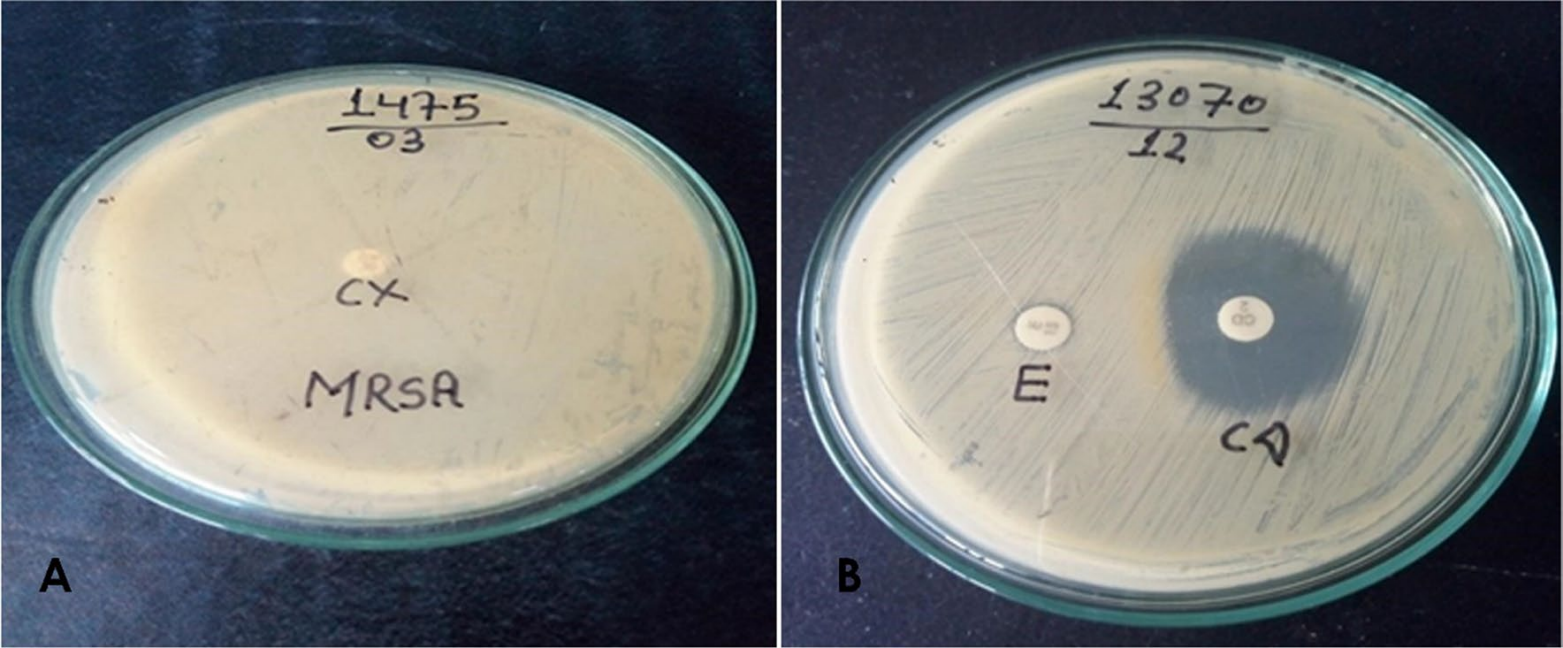
**A** methicillin-resistant *Staphylococcus aureus* in Mueller–Hinton agar plate with cefoxitin disc (30 μg) **B** *Staphylococcus aureus* showing D-shaped zone of inhibition around clindamycin disc (2 μg) when kept adjacent to erythromycin disc (15 μg) in Mueller–Hinton agar plate (D-zone test positive)

The *S. aureus* showing resistance to erythromycin was further tested for inducible resistance to clindamycin. This was tested by the D-zone test as described in CLSI M100-S28 [29]. Briefly, 0.5 McFarland standard bacterial suspension of *S. aureus* was lawn cultured over the MHA plate (HiMedia Pvt. Ltd., India). Then, the erythromycin disc (15 μg) (HiMedia Pvt. Ltd., India) was placed at a distance of 15 mm from the clindamycin disc (2 μg) (HiMedia Pvt. Ltd., India). The plates were incubated aerobically at 37 °C for 18–24 h. The induction test results were interpreted as three different phenotypes of *S. aureus*. The moderate sensitive (MS) phenotypes, if isolates were resistant to erythromycin (zone diameter ≤ 13 mm) and susceptible to clindamycin (zone diameter ≥ 21 mm) without D-shaped zone. The iMLSB phenotypes, if isolates were resistant to erythromycin (zone diameter ≤ 13 mm) and susceptible to clindamycin (zone diameter ≥ 21 mm) with a D-shaped zone. (Fig. 1B) The cMLSB phenotypes, if isolates were resistant to both erythromycin (zone diameter ≤ 13 mm) and clindamycin (zone diameter ≤ 14 mm) [29]. *S. aureus* ATCC 25923 was used as quality control. Separate in-house *S. aureus* isolates that were confirmed as the clindamycin resistance phenotypes were also used for quality control.

### Statistical analysis

All the generated data were entered and curated by using Microsoft Excel® version 2016 (Microsoft Corporation, USA). All statistical analyses were done in R software© version 4.1.1 (R Core Team, Austria). Descriptive summaries were presented in text and tables. Descriptive statistics were expressed as percentages. The 2 × 2 contingency tables were constructed for the categorical variables and the contingency or association was tested using the chi-square test. The Yates correction was applied in the chi-square test whenever applicable. Pearson’s Phi- coefficient was used to measure the effect size, i.e. the strength of the association between two variables. Cohen’s rules-of-thumb were used to interpret the Phi-coefficient. The p-value of less than 0.05 was considered statistically significant whenever applicable.

## Results

Of 1027 samples, 321 (31.2%) samples were culture positive. Of 321 culture positives, 82 (25.5%) were Gram-positive, and, 38 (46.4%) were *S. aureus*. (Table 1).

**Table 1:** Distribution of *S. aureus* isolates

	Culture positive (%)	Gram-positive bacteria (%)	*S. aureus* (%)	MRSA (%)
Clinical specimen				
Urine (n = 859)	225 (26.0%)	22 (9.8%)	8 (3.5%)	3 (37.5)
Pus (n = 52)	41 (78.8%)	32 (78.5%)	16 (39.2%)	8 (50%)
Blood (n = 50)	21 (42%)	9 (42.9%)	4 (19.1%)	1 (25%)
Sputum (n = 41)	23 (56.1%)	14 (60.9%)	6 (26.1%)	2 (33.3%)
*Body fluids (n = 25)	17 (68.0%)	5 (29.41%)	4 (23.5%)	1 (25%)
Hospital care for patients				
Outpatient care (n = 752)	215 (28.5%)	49 (22.8%)	21 (9.8%)	8(38.0%)
Inpatient care (n = 275)	107 (38.9%)	33 (12%)	17 (15.8%)	7 (41.1%)
Sex of patients				
Male (n = 400)	109 (27.3%)	36 (33.0%)	18 (16.5%)	9 (50%)
Female (n = 627)	212 (33.81%)	46 (21.7%)	20 (9.4%)	6 (30%)
Total (n = 1027)	321 (31.3%)	82 (25.5%)	38 (11.8%)	15 (39.4%)

*S. aureus*; *body fluids = CSF, synovial fluid, pleural fluid, throat swabs, vaginal swabs; percentage calculated on respective row total of preceding columnsIn susceptibility testing, all isolates of *S. aureus* were susceptible to vancomycin and 33 (86.8%) were susceptible to amikacin. All isolates were resistant to penicillin-G and ampicillin (Table 2). Also, 25 (67.5%) of *S. aureus* were MDR. Of 38 *S. aureus*, 19 (50%) were screened as MRSA, of which, 15 (39.4%) were confirmed MRSA phenotypically

*MRSA* methicillin-resistant

**Table 2:** Antibiotic resistance profile of *S. aureus*

Class of antibiotics	Antibiotics	Susceptibility (%)	Resistance (%)
Aminoglycosides	Amikacin	33 (86.8)	5 (13.2)
	Gentamicin	28 (73.7)	10 (26.3)
Cephems	Cefoxitin	23 (60.5)	15 (39.5)
	Ceftaroline	30 (78.9)	8 (21.1)
Fluoroquinolones	Norfloxacin	25 (65.8)	13 (34.2)
Folate pathway antagonists	Co-trimoxazole	14 (36.8)	24 (63.2)
Glycopeptides	Vancomycin	38 (100)	0 (0)
Lincosamides	Clindamycin	29 (76.3)	9 (23.7)
Macrolides	Erythromycin	20 (52.6)	18 (47.4)
Penicillins	Ampicillin	0 (0)	38 (100)
	Penicillin-G	0 (0)	38 (100)
	Oxacillin	23 (60.5)	15 (39.5)
Tetracyclines	Tetracycline	20 (52.6)	18 (47.4)

Percentage calculated on *n* = 38

In the induction test, 14 (36.5%) *S. aureus* were iMLSB phenotypes and 7 (18.5%) were cMLSB phenotypes. The cMLSB and iMLSB phenotypes were nearly similar in MRSA and methicillin-susceptible *Staphylococcus aureus* (MSSA) phenotypes. There was no significant association between cMLSB phenotypes and methicillin resistance in *S. aureus* (chi-square with Yates correction (df = 1, *N* = 38) = 0.05, *p* = 0.822). Also, there was a positive, but a very weak effect size (Phi-coefficient = 0.04). Similarly, there was no significant association between iMLSB phenotypes and methicillin resistance in *S. aureus* (chi-square (df = 1, *N* = 38) = 0.11, *p* = 0.745). Also, there was a positive but a very weak effect size (Phi-coefficient = 0.05). (Table 3).

**Table 3:** D-zone test profile of *S. aureus*

Phenotypes	MRSA (%)	MSSA (%)	Chi-square statistic	p-value
cMLSB (EM-R, CD-R)	3 (20%)	4 (17.4%)	0.05*	0.822
iMLSB (EM-R, CD-S, D^ +^)	6 (40%)	8 (34.9%)	0.11	0.745
MS (EM-R, CD-S, D^−^)	2 (13.3%)	5 (21.7%)	0.05*	0.822
Susceptible (EM-S, CD-S)	4 (26.7%)	6 (26%)	0.11*	0.736
Total	15 (100%)	23 (100%)		

*MRSA* methicillin-resistant *Sthaphylococcus aureus*, *MSSA* methicillin-susceptible *Staphylococcus aureus*, *MLSB* macrolide lincosamide-streptogramin B family of antibiotics, *cMLSB* constitutive MLSB phenotype, *iMLSB* inducible MLSB phenotype, *MS* macrolide-streptogramin B phenotype, *EM* erythromycin, *CD*  clindamycin, *R* resistant, *S* susceptible, *D*^+^ D-zone test positive, *D*^−^ D-zone test negative, *Represent the chi-square value with Yates correction

## Discussion

*S. aureus* are one of the most common skin colonizing bacteria, and are a leading source of nosocomial and community-acquired skin infections. Of 1027 samples, 11.8% yielded *S. aureus*. *S. aureus* was higher in pus samples (39.2%). *S. aureus* is normally found in the environment and skin surface, so it is common in wound swabs and pus samples.

In susceptibility testing, all 38 isolates of *S. aureus* were sensitive to vancomycin. All 38 isolates of *S. aureus* were resistant to penicillin-G and ampicillin. Out of 38 *S. aureus*, 67.5% were MDR, and 50% were MRSA. Similar findings were reported by studies in Nepal [21, 23]. Higher percentages were reported by studies in Nepal [14, 22, 24, 26] while lower percentages were reported by other studies in Nepal [20, 25]. The emergence of resistance to multiple antibiotics among *S. aureus* is impeding their effective management. This persuades the physicians to opt for the use of reserve drugs, like MLSB family. Lower cost, lower side effects, and better tissue penetration make clindamycin a better choice among MLSB family. Clindamycin has been used for the treatment of severe staphylococcal infections, like MRSA. During clindamycin therapy, the iMLSB strains can gradually develop constitutively resistant mutants both, in vitro and in vivo. This leads to treatment failures in certain patients. Hence, the detection of such resistant phenotypes is important to minimize treatment failure. *S*. aureus resistance to macrolides may be constitutive or inducible clindamycin resistance, or may solely be macrolides-resistant [10]. However, among erythromycin-resistant *S. aureus* isolates there has been a rise in inducible clindamycin resistance [18]. In this study, about a third of *S. aureus* (36.5%) were iMLSB phenotypes. Similar findings were reported by studies in Nepal [14, 26]. However, lower percentages were reported by other studies in Nepal [20–25] and elsewhere [15–18]. Likewise, 18.5% of *S. aureus* were cMLSB phenotypes. Similar findings were reported by different studies in Nepal [14, 26] and elsewhere [15, 16]. However, lower percentages were reported by different studies in Nepal [24, 26]. Higher percentages were reported by different studies in Nepal [20, 22] and elsewhere [17, 18]. Also, 18.5% of *S. aureus* were MS phenotypes. Similar findings were reported by studies in Nepal [20, 23] while higher percentages were reported by other studies in Nepal [23, 26]. These variances in the reporting of MLSB resistance among *S. aureus* might be related to changes in circulating clones, as well as disparities in infection prevention measures and antibiotic prescribing trends in different hospital settings. Also, the prevalence of MLSB antibiotic-resistant phenotypes varies based on the geography and the characteristics of subjects, like inpatients or outpatients, hospital or community origin, children or adults, public or private institutions, patients or healthcare workers. This emphasizes the need for surveillance programs at nation, region, or hospital level [15–17, 20].

The reporting of MRSA has dramatically risen in recent years. However, there is a stark variation in its reporting among the countries. Inadequate infection-prevention practices in hospitals, indiscriminate antibiotic use, intravascular catheterization, hospitalization in intensive care units, and other factors all contribute to MRSA rise [31]. In this study, 39.4% of *S. aureus* isolates were MRSA. Similar findings were reported by studies in Nepal [21] and elsewhere [17, 18]. However, higher percentages were reported by other studies in Nepal [22, 23, 26] and elsewhere [11, 14]. And, lower percentages were reported by other studies in Nepal [12, 20, 25] and elsewhere [15, 16]. These variances in MRSA reporting among *S. aureus* isolates might be related to changes in circulating clones in a different geography, as well as disparities in infection- prevention measures and antibiotic prescribing trends in different hospital settings.

In this study, there was no substantively significant association between cMLSB resistance and methicillin resistance in *S. aureus* (chi-square with Yates correction (df = 1, *N* = 38) = 0.05, *p* = 0.822, Phi-coefficient = 0.04). Also, there was no substantively significant association between iMLSB resistance and methicillin resistance in *S. aureus* (chi-square (df = 1, *N* = 38) = 0.11, *p* = 0.745, Phi-coefficient = 0.06). Genotyping i.e., detection of the erm genes is considered a superior tool for surveillance of MLSB resistance. But the continuous mutations in erm genes make the use of genotyping tools difficult. Also, the use of such expensive tools in poor resource settings is hypothetical. In contrast, the phenotypic techniques indicate both the presence and expression of the responsible erm gene. The D-zone test is a simple and cheap phenotypic technique using erythromycin and clindamycin discs. This is a phenotypic disc diffusion test recommended by CLSI [29]. The D-zone test relies on the ability of erythromycin to induce resistance against clindamycin. A flattening of the zone of inhibition around the clindamycin disc in the proximity of the erythromycin disc, producing a D-shaped zone of inhibition, is considered a positive D-zone result. This indicates the induction of clindamycin resistance by erythromycin. The D-zone test has a high throughput reporting different types of phenotypic resistance in a single test. This method has a sensitivity of 100% when the distance of two test disk is 15 mm [32].

Globally, AMR is on the rise, particularly in developing countries, like Nepal. Over the counter sale of antibiotics, lack of effective regulations on antibiotics use, incomplete dosing, excessive use of wide-spectrum antibiotics for common infections, and empiric therapy without laboratory diagnosis are all common in developing countries like Nepal. These practices usually cure infections, so most health settings opt for and retain these practices, but in return, these settings act as a factory of resistant mutants. This, in part, is because of a lack of sufficient resources to set up standard laboratory facilities covering all geography, particularly in remote skirts of developing countries like Nepal. AMR is a public health threat that demands urgent attention. Surveillance of this type reports the updated AMR profile of the circulating pathogens in the region, which in turn can be used for formulating policies with strong strategies to check AMR.

## Limitations

First, the selection bias could have accounted for some errors in the findings. Second, the study was conducted for six months and the total samples were 1027 in a single setting. This is a comparatively small sample size over the shorter duration. Thus, the generalization of the findings may not be accurate. However, we believe that the medical practices and patient distributions in every hospital in the capital city, Kathmandu, are more or less similar, excluding the specialized hospitals. Thus, we believe our findings represent the population scenario of the region. Third, the study design consisted only of the phenotypic characterization, which could be the result of many intrinsic and extrinsic factors. The genotypic characterization could have provided further clear insights.

## Conclusions

The high proportions of MRSA and iMLSB phenotypes among *S. aureus* emphasize the need for methicillin resistance test and D-test to be incorporated in routine susceptibility testing for effective management of *S. aureus*. This also warrant the need for larger epidemiological AMR surveillances and policy updates to manage *S. aureus* effectively.

## Data Availability

The complete dataset generated and analyzed during the study is already covered in the text. The raw data can be made available at reasonable request to the corresponding author.

## Abbreviations

CD: Clindamycin
CLSI: Clinical and Laboratory Standards Institute
cMLSB: Constitutive MLSB phenotype
D^−^: D-zone test negative
D^+^: D-zone test positive
EM: Erythromycin
iMLSB: Inducible MLSB phenotype
MDR: Multidrug-resistant
MHA: Mueller–Hinton agar
MLSB: Macrolide lincosamide-streptogramin B family of antibiotics
MRSA: Methicillin-resistant *Staphylococcus aureus*
MS: Macrolide-streptogramin B phenotype
MSSA: Methicillin-susceptible *Staphylococcus aureus*
R: Resistant
S: Susceptible
